# Comparing Different Montages of Transcranial Direct Current Stimulation in Treating Treatment-Resistant Obsessive Compulsive Disorder: A Randomized, Single-Blind Clinical Trial

**DOI:** 10.3390/medicina61020169

**Published:** 2025-01-21

**Authors:** Che-Sheng Chu, Yen-Yue Lin, Cathy Chia-Yu Huang, Yong-An Chung, Sonya Youngju Park, Wei-Chou Chang, Chuan-Chia Chang, Hsin-An Chang

**Affiliations:** 1 Department of Psychiatry, Kaohsiung Veterans General Hospital, Kaohsiung 813, Taiwan; cschu@vghks.gov.tw; 2 Center for Geriatrics and Gerontology, Kaohsiung Veterans General Hospital, Kaohsiung 813, Taiwan; 3 Non-Invasive Neuromodulation Consortium for Mental Disorders, Society of Psychophysiology, Taipei 114, Taiwan; 4 Graduate Institute of Medicine, College of Medicine, Kaohsiung Medical University, Kaohsiung 807, Taiwan; 5 Department of Emergency Medicine, Tri-Service General Hospital, National Defense Medical Center, Taipei 114, Taiwan; yyline.tw@yahoo.com.tw; 6 Department of Emergency Medicine, Taoyuan Armed Forces General Hospital, Taoyuan 325, Taiwan; 7 Department of Life Sciences, National Central University, Taoyuan 320, Taiwan; chuang@ncu.edu.tw; 8 Department of Nuclear Medicine, College of Medicine, The Catholic University of Korea, Seoul 296-12, Republic of Korea; yongan@catholic.ac.kr (Y.-A.C.); park@catholic.ac.kr (S.Y.P.); 9 Department of Radiology, Tri-Service General Hospital, National Defense Medical Center, Taipei 114, Taiwan; weichou.chang@gmail.com; 10 Department of Psychiatry, Tri-Service General Hospital, National Defense Medical Center, Taipei 114, Taiwan

**Keywords:** electroencephalogram, functional connectivity, obsessive compulsive disorder, pre-supplementary motor area, transcranial direct current stimulation

## Abstract

*Background*: Transcranial direct current stimulation (tDCS) is a non-invasive brain stimulation for treatment-resistant obsessive compulsive disorder (OCD). We aim to compare the treatment outcomes of a newly developed dual-site cathodal tDCS method over the orbitofrontal cortex (OFC) and pre-supplementary motor area (pre-SMA) and two previously reported montages (cerebellum-OFC and pre-SMA) in patients with treatment-resistant OCD. *Methods*: Eighteen OCD patients were randomly assigned to receive twice-daily 2 mA/20 min sessions for 10 consecutive weekdays, with the active cathode placed on the cerebellum-OFC, bilateral pre-SMA, or OFC-pre-SMA tDCS. The primary outcome was the change in the Yale–Brown Obsessive Compulsive Scale (Y-BOCS). The resting electroencephalogram (EEG) was recorded to obtain the default mode network (DMN) via low-resolution electromagnetic tomography. Each patient received one-week and one-month follow-ups after two weeks of stimulation. *Results*: At the end of the stimulation, the Y-BOCS scores in the cerebellum-OFC, pre-SMA, and OFC-pre-SMA tDCS groups (n = 6 in each group) were decreased by 14.15 ± 13.31, 7.4 ± 9.59, and 20.75 ± 8.70%, respectively, but no significant differences were found among the groups. In the OFC-pre-SMA tDCS group, OC symptoms significantly decreased by a mean of −20.75% immediately after the 20th tDCS session, and the improvement remained at 1 week and 1 month after tDCS. EEG source functional connectivity analyses revealed increased functional connectivity within the frontal network after OFC-pre-SMA tDCS, whereas decreased functional connectivity within the DMN was observed after cerebellum-OFC tDCS. *Conclusions*: Dual-site cathodal tDCS over the OFC and pre-SMA might be considered a potential montage to treat patients with treatment-resistant OCD. Future studies using randomized sham-controlled designs are needed.

## 1. Introduction

Obsessive compulsive disorder (OCD) is characterized by distressing obsessions with recurrent and persistent thoughts, urges, or images and compulsions of repetitive behaviors or mental acts aiming to prevent or reduce anxiety or distress. Obsessions and compulsions are time-consuming and can cause significant functional impairment, according to the Diagnostic and Statistical Manual of Mental Disorders, Fifth Edition (DSM-5) criteria [1]. OCD typically starts between childhood and early adulthood and is a common chronic and disabling disorder with high proportions of treatment resistance. Around one-third of OCD patients fail to benefit significantly from first-line treatment, such as serotonin reuptake inhibitors (SSRIs) or cognitive behavioral therapy [2]. Combining treatments, augmenting with antipsychotics, and switching strategies as second-line treatments also fail to bring much benefit for these patients. Given the high proportion of OCD patients not responsive to the available first- and second-line treatments, the development of novel treatment strategies using non-invasive brain stimulation are being considered. For example, the US Food and Drug Administration has approved the use of the deep transcranial magnetic stimulation (TMS) H7 coil for treatment-resistant OCD [3].

Transcranial direct current stimulation (tDCS) is a promising, safe, tolerable, easy-to-use, and cost-effective NIBS, although there is little evidence of its effectiveness for OCD. tDCS applies a weak direct current on the scalp and through the brain, rapidly leading to shifts in the resting membrane potentials that may alter cortical excitability and the function of neural systems. The facilitation of depolarization or hyperpolarization of cortical pyramidal cell somas by the radial current flow contributes to increased or decreased cortical excitability of the brain regions under the anode or cathode [4]. tDCS has local effects on the modulation of excitation–inhibition balance at the stimulation site, as well as physiological effects at the network level [5]. Simultaneously, applying tDCS over different areas could modulate brain activity and connectivity within large, widespread brain networks. For example, facilitatory anodal tDCS over the primary motor cortex (M1) with inhibitory cathodal tDCS of the contralateral frontopolar cortex resulted in significantly increased functional connectivity within premotor, motor, and sensorimotor areas of the simulated hemisphere [6]. Furthermore, cathodal stimulation of the bilateral inferior parietal lobe nodes of the default mode network (DMN) decreased the frequency of negative mind-wandering thoughts about the past [7]. DMN is considered a task-negative network, reflecting a collection of brain regions that deactivate during cognitive processing. Repeated tDCS with inter-session intervals can prolong the after-effects and behavioral effects of tDCS by modifying the efficacy of N-methyl-D-aspartate receptors, and it is increasingly used for therapeutic applications in neuropsychiatric disorders [8]. Taken together, tDCS might alleviate targeted symptoms through altered molecular mechanisms and brain connectivity.

Neuroimaging studies have identified abnormal activity and connectivity within the orbito-fronto-striato-pallido-thalamic network in patients with OCD, including decreased activity in the parietal cortex and cerebellum, as well as increased activity in the supplementary motor area (SMA), anterior cingulate cortex, orbitofrontal cortex (OFC), and the caudate [9]. In a randomized sham-controlled study in 21 patients with treatment-resistant OCD who received 10 sessions (twice daily) of 2 mA for 20 min over a 3-month follow-up period, cerebellum-OFC tDCS applied with the cathode over the orbitofrontal cortex (OFC) and the anode over the right cerebellum induced a significant acute reduction in OCD symptoms immediately after the tDCS regimen compared to sham stimulation [10]. In a randomized sham-controlled study on 43 patients with treatment-resistant OCD who received 20 daily sessions of 2 mA for 30 min over an 8-week follow-up period, pre-SMA tDCS applied with the cathode over the SMA and the anode over the left deltoid induced a significant reduction in OCD symptoms compared to sham stimulation [11]. All published studies using tDCS in OCD patients were based on stimulation in one brain region. Based on multiple circuits (e.g., cortico-striatal-thalamic-cortical (CTSC)) involved in OCD, it is rational to target multiple brain regions when using brain stimulation techniques. For example, rTMS targeting multiple brain regions of the left dorsolateral prefrontal cortex + bilateral SMA alone may benefit from right OFC augmentation stimulation in patients with treatment-resistant OCD with comorbid major depressive disorder [12]. Therefore, we propose that dual-site tDCS, which is related to CTSC, may benefit more than previously used montages, such as cerebellum-OFC and SMA. It has been nearly 20 years since the US Food and Drug Administration last approved oral medication for OCD, and approximately 40 to 60% of OCD patients do not respond well to SSRIs [13]. Therefore, clinical trials are important for the development of new interventional strategies for treatment-resistant OCD patients. Furthermore, given the high variability of tDCS montages as a limitation for OCD treatment, previous studies have applied computer modeling analysis to evaluate the electric field (EF) strengths in specific brain structures associated with OCD symptoms. EF analysis found that montages using the main electrode over the SMA with an extracephalic reference electrode might lead to stronger EFs. Therefore, the application of an extracephalic positioning as a reference electrode might provide better efficacy in reducing OCD symptoms. Future randomized controlled studies are encouraged to investigate better tDCS montages/protocols (e.g., tDCS simultaneously targeting multiple OCD-related structures) in larger sample sizes [14]. To our knowledge, no study has investigated the effects of concurrent dual-site cathodal tDCS over the OFC and pre-SMA (OFC-pre-SMA tDCS) with an extracephalic reference electrode in patients with treatment-resistant OCD.

Considering the involvement of CTSC abnormalities in the pathogenesis of OCD, a study suggested that patients with OCD demonstrate consistently neuropsychological deficits in executive function, processing speed, sustained attention, and non-verbal memory deficits [15]. For example, a study of 40 patients with OCD performing the Tower of London test of planning suggested that patients have a selective deficit in generating alternative strategies when they make a mistake compared to healthy controls [16]. Another study reported executive and working memory deficits in patients with OCD associated with treatment resistance, as measured using the Stroop test [17]. Furthermore, heart rate variability (HRV)—a tool to assess information about alterations in the function of the autonomous nervous system—may be a potential physiological biomarker of generalized anxiety disorder. A study showed patients with OCD who responded to treatment had a higher parasympathetic tone compared to those who did not respond to treatment [18]. Taken together, the application of neuropsychological and neurological tests would allow us to not only understand the cognitive deficit and autonomic nervous system activity in patients with OCD, but to also examine the potential effects of interventions (e.g., tDCS in the present study) in these patients.

The aim of this study was to investigate the effectiveness of OFC-pre-SMA tDCS and another two previously reported tDCS montages (i.e., cerebellum-OFC tDCS and pre-SMA tDCS) in patients with treatment-resistant OCD. The null hypothesis was that there would be no difference in the effectiveness of treating OCD symptoms when comparing the three different tDCS montages. As a secondary aim, the neuropsychological and neurophysiological changes were also measured after the tDCS interventions. We hypothesized that tDCS intervention with three different montages would be associated with distinct changes in these secondary outcomes.

## 2. Materials and Methods

### 2.1. Participants

The trial was approved by the ethics committee of Tri-Service General Hospital, Taipei, registered in Taiwan (ClinicalTrials.gov ID: NCT05595421). We consecutively recruited participants from psychiatric outpatient clinics in the present study. The inclusion criteria were as follows: (1) eligible participants aged 20–65 with a primary clinical diagnosis of DSM-5-defined obsessive compulsive disorder; (2) being treatment-resistant, as revealed by a Yale–Brown Obsessive and Compulsive Scale score (Y-BOCS) of >16 despite at least two SSRI trials of adequate dose and duration (or refusal to take medication for personal choice) and having been offered prior cognitive behavior therapy by a trained practitioner; (3) a stable psychopharmacological medication dosage, with no changes for 1 month before the beginning of the trial and remaining unchanged throughout the entire duration of the study; and (4) agreement to participate in the study and provide written informed consent. The exclusion criteria were as follows: (1) having major mental disorders except OCD based on DSM-5 criteria; (2) having contraindications for transcranial electrical stimulation or transcranial magnetic stimulation, e.g., pacemakers, metallic or magnetic pieces in the head/brain, ear implants, or other implantable brain medical devices; (3) pregnancy or breastfeeding at enrollment; (4) having active substance use disorder (with the exception of caffeine and/or tobacco); (5) having a history of seizures; (6) having a history of intracranial neoplasms or surgery, or a history of severe head injuries or cerebrovascular diseases; (7) skin lesions on the scalp at the area of electrode application; or (8) having unstable medical conditions at enrollment. Participants received compensation of USD 6.67 if they properly completed the study.

### 2.2. tDCS Intervention

**Figure 1:**
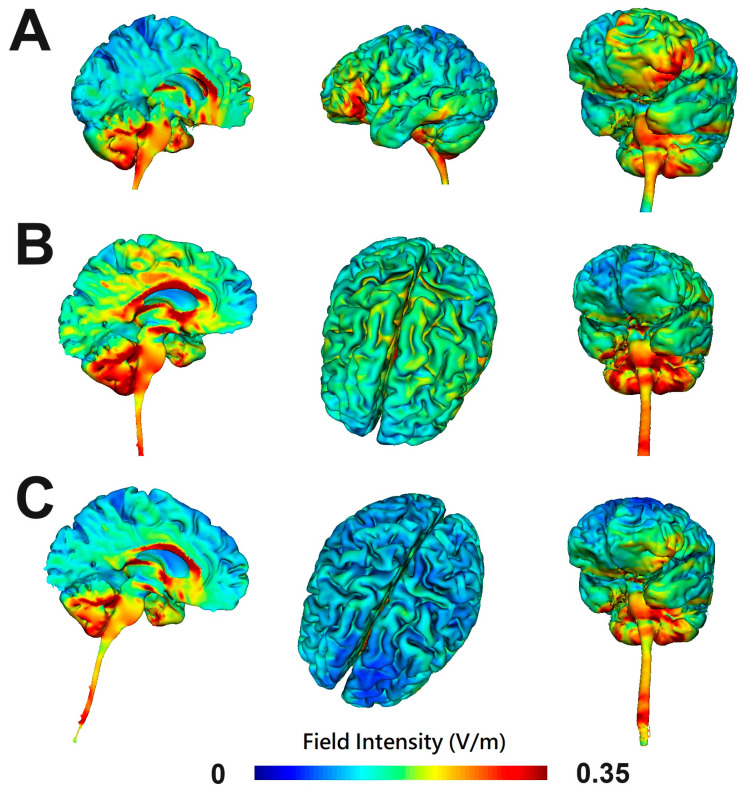
Three-dimensional representations of electric field simulation for different montages of tDCS with a current intensity of 2 mA: (**A**) cerebellum-OFC tDCS (anode over the right cerebellum and cathode over the left OFC), (**B**) pre-SMA tDCS (cathode over the bilateral pre-SMA and reference electrode over the lateral surface of the right deltoid), and (**C**) OFC-pre-SMA tDCS (the first cathode over the pre-SMA, the second cathode over left OFC, and the reference electrode over the lateral surface of the right deltoid). The computational models of the cortical electric fields were estimated using HD-Explore^®^ software version 7.0.0. (Soterix Medical, New York, NY, USA), which utilizes a finite element model of brain current flow based on an MRI-derived MNI 152 template head. OFC, the orbitofrontal cortex; pre-SMA, pre-supplementary motor area.

This study was a randomized, single-blind, clinical trial comparing different montages of transcranial direct current stimulation on treatment-resistant obsessive compulsive disorder. The participants were randomized into one of the three groups: (1) cerebellum-OFC tDCS, (2) pre-SMA tDCS, and (3) OFC-pre-SMA tDCS. Figure 1 illustrates the modeling of electric field distribution across the three tDCS groups. Stimulation sessions were applied using a DC stimulator (neuroConn, Ilmeneau, GmbH). All patients received twice-daily sessions separated by at least 1 h for 10 consecutive weekdays. We chose twice-daily stimulation instead of daily simulation because of previous studies showing better efficacy with twice-daily stimulation compared to once a day. For example, in a double-blind randomized study of 93 vascular depression, receiving tDCS stimulation for 10 consecutive working days showed that twice-daily stimulation achieved significantly greater symptom improvement than once-daily stimulation and sham stimulation [19]. Furthermore, twice-daily tDCS stimulation boosted cortical plasticity and improved memory in a study of 124 patients with Alzheimer’s disease [20]. For treatment-resistant OCD, a randomized, sham-controlled, double-blind study demonstrated that OCD patients who received twice-daily anodal tDCS stimulation over pre-SMA had a significantly greater response rate than those with sham stimulation [21]. Therefore, we chose twice-daily sessions in the present study. Each session of tDCS consisted of a direct current of 2 mA delivered for 20 min. In the cerebellum-OFC tDCS group, two 7 cm × 5 cm (35 cm^2^) sponge electrodes soaked in a saline solution (0.9% NaCl) were applied with the anode placed over the right cerebellum (i.e., 3 cm below the inion and 1 cm right from the midline) and the cathode over FP1, according to the international 10–20 system (i.e., the left OFC). In the pre-SMA tDCS group, the cathode (5 cm × 5 cm) was placed on the sagittal midline at 15% of the distance between the inion and nasion anterior to the Cz (vertex) to target the bilateral pre-supplementary motor area (pre-SMA). The reference electrode (5 cm × 7 cm) was placed on the lateral surface of the patient’s right deltoid. In the OFC-pre-SMA tDCS group, the stimulator was connected to a 2 × 1 wire adaptor (Equalizer Box, NeuroConn). The first cathode (5 cm × 5 cm) was placed over the sagittal midline at 15% of the distance between the inion and nasion anterior to the Cz (vertex). The second cathode (5 cm × 7 cm) was placed over FP1. The reference electrode (5 cm × 7 cm) was placed on the lateral surface of the right deltoid. The study outcomes were measured using a clinical rater (HAC) blinded to the group assignment. The adverse effects of tDCS were measured using a well-established tool [22]. The timeline for treatment and assessments is provided in Table 1. For the primary outcome (Y-BOCS) and mood measurement (e.g., HAM-D), each participant received four assessments (at baseline, at the end of stimulation, and at the 1-week and 1-month follow-ups). For the cognitive measurements and neurophysiological tests (HRV and EEG), each participant received two assessments (at baseline and the end of stimulation).

### 2.3. Clinical Outcome Measures

The primary outcome measure was the change in the score of the Y-BOCS from baseline to the end of stimulation. Y-BOCS is a 10-item scale designed to assess obsessions and compulsions by measuring the severity and type of symptoms of OCD patients over the past seven days. Total Y-BOCS scores range from 0 to 40, with higher scores indicating greater severity of OCD symptoms. Scores on the obsession and compulsion subscales range from 0 to 20, but only the total Y-BOCS score is interpreted [23]. Other clinical measures included the changes from baseline in the scores of the self-reporting OCD Visual Analog Scale (OCD-VAS), Hamilton Depression Rating Scale (HAM-D), Hamilton Anxiety Rating Scale (HAM-A), and World Health Organization Quality of Life-BREF (WHO-BREF). We included the HAM-D, HAM-A, and WHO-BREF because patients with OCD tend to experience comorbid depression and anxiety symptoms, which may both contribute to a lower quality of life [24].

### 2.4. Neuropsychological Outcomes

Neuropsychological outcomes were measured with the Color Trails Test (CTT) for assessing sustained visual attention [25], the Stroop Color Word Test (SCWT) for assessing selective attention and cognitive flexibility [26], Connors’ Continuous Performance Test, 2nd Edition (CPT-II), for assessing concentration, sustained attention, response inhibition and impulsivity [27], the Tower of London–Drexel University Test, 2nd Edition (TOLDXtm) [28], for assessing executive functioning, especially concerning the ability of planning, processing, and problem-solving skills, and the Digit Symbol Substitution Test (DSST) for assessing attention, executive function, and processing speed. OCD patients appear to have wide-ranging cognitive deficits, particularly in executive function, verbal memory, verbal fluency [29], and attention [30]. Therefore, we chose several cognitive tests covering different domains of cognitive deficit in the present study.

### 2.5. Neurophysiological Outcomes

#### 2.5.1. Heart Rate Variability (HRV)

The participants sat quietly in a light-controlled, soundproof room and breathed spontaneously. After a 20 min rest, two electrodes were placed on the right and left arm just below the elbow and a ground electrode placed below the wrist on the right arm with a lead I electrocardiogram taken for 5 min. An HRV analyzer (LR8Z11, Yangyin Corp., Taipei, Taiwan) acquired, stored, and processed the electrocardiography signals. Power spectral analysis was performed using non-parametric fast Fourier transformation to obtain the high-frequency power (HF, 0.15–0.40 Hz), which represents the vagal control of HRV.

#### 2.5.2. Electroencephalogram (EEG)

The resting-state EEG was collected using a 19-channel EEG (Neuro Prax^®^ TMS/tES compatible full-band DC-EEG system, NeuroConn GmbH, Ilmenau, Germany) with Ag/AgCl sintered ring electrodes placed according to the international 10–20 system, using a sampling frequency of 4000 Hz, analog–digital precision of 24 bits, and an analogous 0–1200 Hz bandpass filter. The patients sat comfortably in a recliner in a light- and sound-attenuated room. The participants were instructed not to drink caffeinated beverages one hour before the EEG recording or alcohol 24 h before the recording to avoid caffeine- or alcohol-induced changes in the EEG stream.

The reference and ground electrodes were placed at the tip of the nose and Fpz, respectively. The horizontal electrooculogram, recorded using two electrodes, was placed at 1 cm from the outer canthi of both eyes. Two electrodes were placed above and below the left eye, respectively, to record the blinks and vertical electrooculogram. The impedance of each electrode was checked to ensure it remained below 5 kΩ. The patients were instructed to stay relaxed and in a state of mind-wandering with their eyes closed, and the resting-state EEG was recorded for 5 min. Offline, the data were down-sampled to 256 Hz, band-pass-filtered to 1–100 Hz, and analog 60 Hz notch-filtered using EEGLAB v2020.0 [31]. To avoid removing meaningful neural signals, we conducted artifact subspace reconstruction techniques, which is an adaptive method for the correction of artifacts. It detects and reconstructs the subspace of the EEG data contaminated by artifacts. This method leverages the statistical properties of the EEG signal to differentiate between neural activity and artifacts. By identifying the subspace where artifacts dominate, artifact subspace reconstruction can eliminate the artifacts signal [32]. Independent component analysis (ICA), followed by ICLabel [33], was used to automatically remove artifacts caused by muscle activity, heartbeats, eye movements, and eye blinks. Only accepted epochs of eyes-closed rsEEG data were chosen for electrical source estimation.

#### 2.5.3. Functional Connectivity Analysis in EEG Source Space

EEG source estimation and connectivity analysis were performed using the exact low-resolution electromagnetic tomography (eLORETA) algorithm, a linear inverse solution for scalp-recorded EEG signals that have no localization error for point sources under noise-free conditions [34]. Cortical regions of interest (ROIs) for connectivity analysis were determined a priori based on previous studies reporting dysfunctional connectivity within the default mode network (DMN) and frontal network (FN) in OCD [35]. A voxel-wise approach was adopted to determine the ROIs (4 from the DMN and 5 from the FN) by including all gray matter voxels within a 20 mm radius of the seed points (see their MNI coordinates in Table 2). Time series of the average current density of all voxels from each ROI were calculated for the subsequent computation of functional connectivity. Functional connectivity between each pair of ROIs was defined as the lagged non-linear coherence of intracortical EEG source estimates. Only the lagged coherence was considered as physiological connectivity between the ROIs because instantaneous (or zero-lag) coherence can be caused by intrinsic artifacts due to the low spatial resolution of the eLORETA solution and non-physiological effects, such as volume conduction [34]. Only the non-linear part of lagged coherence was considered because this measure was based on normalized Fourier transforms and did not consider any amplitude information. Previous research has linked non-linear lagged coherence with a fundamental process of neural communication associated with synaptic plasticity [36]. The lagged non-linear coherence was calculated for the EEG delta (1.5–4 Hz), theta (4–8 Hz), alpha (8–12 Hz), beta 1 (12–30 Hz), beta 2 (20–30 Hz), and gamma (30–80 Hz) frequency bands.

### 2.6. Sample Size Calculation

The sample size was estimated using data from previous research on tDCS in treating OCD to obtain a comparison between the baseline and endpoints of the active treatment group [9]. In an open-label trial by Bation et al., cerebellum-OFC tDCS largely improved OCD symptoms (effect size = 1.21) [37]. In a randomized controlled trial by Da Silva et al., pre-SMA tDCS also largely improved OCD symptoms (effect size = 1.05 for the active group) [11]. In this trial, OFC-pre-SMA tDCS was assumed to have additive treatment effects of cerebellum-OFC tDCS and pre-SMA tDCS. With these assumptions, a large difference was estimated between the tDCS interventions with three different montages. G*power 3.1.9.4 is used to calculate the sample size of ANOVA (fixed effects, omnibus, one-way) for the primary outcome measurement with an effect size f = 0.6, alpha error probability = 0.05, power (1 −beta error probability) = 0.8, and number of groups = 3. The calculated total sample size was 30. Thus, a sample of 36 (N = 12 in each group; please see ClinicalTrials.gov ID: NCT05595421) was required to assume a 16% dropout rate. The trial stopped before it attained its planned sample size as the researchers failed to enroll eligible participants in time and, consequently, funding was discontinued.

### 2.7. Statistics

The SPSS Statistics 24.0 software (IBM SPSS Inc., Chicago, IL, USA) was used for all analyses. Univariate analyses of variance (ANOVAs) were conducted to compare the demographic data, the clinical variables at baseline, and the changes in the outcome measures after tDCS among the three tDCS groups. Chi-squared tests were used to examine between-group differences in discrete variables. The null hypothesis was rejected at *p* < 0.05 for the primary outcome measure (i.e., the change in the Y-BOCS score from the baseline to the end of stimulation). A significant effect would be followed by post hoc analyses, with Bonferroni corrections applied for multiple tests. For the within-group comparisons of continuous variables, paired *t*-tests were used for the parametric variables, and Wilcoxon signed-rank tests were used for non-parametric variables. In these analyses, we did not apply corrections for multiple comparisons due to the nature of exploratory testing. Paired *t*-tests were used to compare the EEG source functional connectivity between each ROI (in each frequency band) at the end of stimulation to that at baseline. The statistical non-parametric mapping methodology included in the eLORETA software v20201109 was used to adjust for multiple comparisons by implementing a non-parametric permutation procedure (5000 randomizations). The level of significance for the analyses conducted was set at *p* = 0.05 (two-tailed). Access to the dataset or code used in this study will be shared upon reasonable request.

## 3. Results

### 3.1. Sample Characteristics

Of the 64 screened patients, 41 were eligible. Only 18 agreed to participate in the trial and were randomly assigned to receive cerebellum-OFC (n = 6), pre-SMA (n = 6), or OFC-pre-SMA tDCS (n = 6). All of them completed 20 sessions of the trial. There were no significant between-group differences in the sociodemographic and clinical characteristics at baseline (Table 3). No participant dropped out of this study.

### 3.2. Primary Outcome

**Figure 2:**
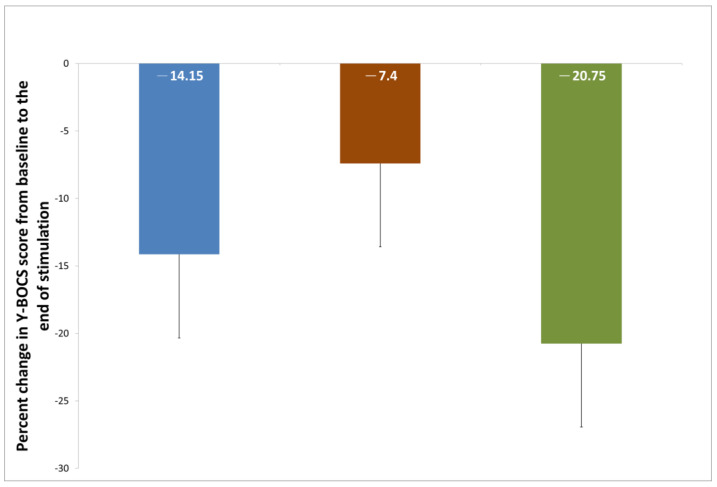
Percent change in Y-BOCS score from baseline to the end of stimulation across three tDCS groups.

At the end of stimulation, the percentages of Y-BOCS score reduction in the cerebellum-OFC, pre-SMA, and OFC-pre-SMA tDCS groups were 14.15 ± 13.31, 7.4 ± 9.59, and 20.75 ± 8.70%, respectively. When comparing the three groups, there were no significant differences in the percentage changes in the Y-BOCS score at the end of stimulation (F = 2.32, *p* = 0.13, Figure 2). The results were similar at the one-week follow-up (F = 0.61, *p* = 0.56) and the one-month follow-up (F = 0.13, *p* = 0.88). Regarding the difference in the Y-BOCS raw score from the baseline, within-group analyses showed that the Y-BOCS score was significantly reduced at the end of stimulation in the cerebellum-OFC (t = −2.70, *p* = 0.043) and OFC-pre-SMA (t = −4.68, *p* = 0.005) tDCS groups. The effects in both groups persisted until the end of the trial (all *p* values < 0.05). The reduction in the Y-BOCS score was not significant at the end of stimulation in the pre-SMA tDCS group (t = −1.90, *p* = 0.12) but reached significance at the one-week (t = −2.65, *p* = 0.045) and one-month (t = −2.62, *p* = 0.047) follow-ups. When comparing the three groups, there were no significant differences in the changes in the Y-BOCS raw score at the baseline, the end of stimulation (F = 1.63, *p* = 0.23), the one-week follow-up (F = 0.88, *p* = 0.48), or the one-month follow-up (F = 0.20, *p* = 0.82).

### 3.3. Secondary Outcomes

The changes in the scores of the HAM-D, HAM-A, OCD-VAS, and all WHOQOL-BREF subdomains at the end of stimulation and follow-up visits were not significantly different among the cerebellum-OFC, pre-SMA, and OFC-pre-SMA tDCS groups (all *p* values > 0.05). Within-group analyses showed that compared to baseline, OCD-VAS scores at the end of stimulation (t = −4.70, *p* = 0.005), at the one-week follow-up (t = −3.89, *p* = 0.011), and at the one-month follow-up (t = −2.70, *p* = 0.043) were significantly reduced in the OFC-pre-SMA tDCS group. The psychological domain score of WHOQOL at the one-month follow-up (t = 2.99, *p* = 0.031) was significantly increased in the pre-SMA tDCS group. When comparing the three groups, there were no significant differences in the change in the cognitive test performance or HF-HRV at the end of stimulation (F = 0.11, *p* = 0.90). Within-group analyses showed that compared to the baseline, the CTT Color 2 time (t = 5.91, *p* = 0.002) and CPT-II d’ (z = 2.20, *p* = 0.03) at the end of stimulation were significantly decreased and increased in the pre-SMA tDCS group.

### 3.4. Safety and Tolerability

tDCS was well tolerated by the patients, and no serious adverse events were observed.

There were no significant differences in the occurrences of treatment-emergent adverse events among the three tDCS groups (Table 4).

### 3.5. EEG Source Functional Connectivity Analyses

**Figure 3:**
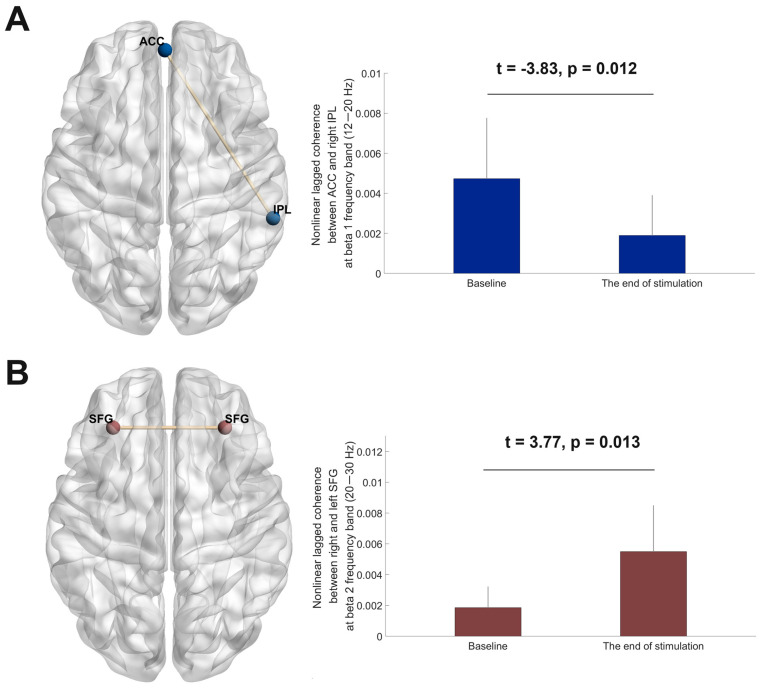
(**A**) The cerebellum-OFC tDCS (anode over the right cerebellum and cathode over the left OFC) group had significant decreases in the beta-1 band (12–20 Hz) non-linear lagged coherence within the default mode network (DMN) from the baseline to the end of stimulation, specifically between the anterior cingulate cortex (ACC) and right inferior parietal lobule (IPL). (**B**) The OFC-pre-SMA tDCS (the first cathode over the pre-SMA, the second cathode over left OFC, and the reference electrode over the lateral surface of the right deltoid) group had significant increases in the beta-2 band (20–30 Hz) non-linear lagged coherence within the frontal network (FN) from the baseline to the end of stimulation, specifically between the right and left superior frontal gyrus (SFGs). The figure was created using eLORETA and BrainNet Viewer. Error bars indicate standard deviations.

Significant differences between the baseline and the end of stimulation emerged for within-DMN connectivity in the cerebellum-OFC tDCS group. Non-linear lagged coherence between a region in the anterior cingulate gyrus and a region in the right inferior parietal lobule in the beta 1 frequency band (12–20 Hz) was decreased at the end of stimulation compared to the baseline (t = −3.83, *p* = 0.012; Figure 3A). In the OFC-pre-SMA tDCS group, within-FN connectivity significantly changed from the baseline to the end of stimulation. Non-linear lagged coherence between a region in the right superior/middle frontal gyrus and a region in the left superior/middle frontal gyrus in the beta 2 frequency band (20–30 Hz) was increased at the end of stimulation compared to the baseline (t = 3.77, *p* = 0.013; Figure 3B). Neither within-DMN nor within-FN connectivity significantly changed from the baseline to the end of stimulation in the pre-SMA tDCS group.

## 4. Discussion

Our study is the first to investigate the effectiveness of concurrent dual-site cathodal tDCS over the OFC and pre-SMA (OFC-pre-SMA tDCS) applied as a therapeutic tool to reduce OC symptoms in patients with treatment-resistant OCD. Our study is also the first to compare the clinical effects among three kinds of tDCS montages (cerebellum-OFC, OFC-pre-SMA, and pre-SMA tDCS) in treating OCD.

The present study provides preliminary results showing that the novel dual-site OFC-pre-SMA cathodal tDCS contributed to a higher reduction in OC symptoms than previous commonly adopted montages (cerebellum-OFC and pre-SMA tDCS). The first meta-analysis of eight studies, including four randomized controlled trials (RCTs) and four open-label trials on 241 patients with OCD, showed that the pooling data demonstrated a large effect of tDCS on reducing OCD symptoms, but active tDCS was not superior to sham stimulation at the end of treatment when considering only the RCTs. The high variability of tDCS montages and small sample sizes might explain the lack of a positive effect when comparing active to sham tDCS. Other potential confounding factors, such as variation in participant compliances, the baseline severity of symptoms, or differences in individual neuroanatomy, might affect the tDCS efficacy. The pre-SMA was the most used montage in the meta-analysis study, followed by the cerebellum-OFC and SMA [9]. No dual-site tDCS montage, such as that in the present study, has been applied in the treatment of OCD patients. Compared to a previous meta-analysis study, the present study enrolled patients of comparative ages (34.72 vs. 36.14) but a lower percentage of females (27.8% vs. 53.9%). Given the different study designs and protocols, it is hard to directly compare the results among varies studies. However, for the present study, several factors may explain the higher efficacy of the novel montage of dual-site FOC-pre SMA cathodal tDCS in the treatment of OCD. First, it could induce higher EFs and allow the current flow to penetrate more deeply into the brain [9]. Second, dual-site tDCS montage stimulates more brain regions involved in the CTSC neurocircuitry of OCD, leading to a higher reduction in OC symptoms [38]. Notably, the results should be interpreted with caution because there was no sham control group in the present study. The placebo response should not be overlooked in brain stimulation trials. However, a study showed that a reduced placebo response was observed for OCD compared to other psychiatric diagnoses [39]. The present study enrolled treatment-resistant OCD, which may make the symptoms more resistant to further intervention.

Our results indicate that tDCS using different montages was safe and tolerable in patients with treatment-resistant OCD. Although the improvement of OCD symptoms at the end of stimulation was not significantly different among the three tDCS groups, OFC-pre-SMA tDCS was considered the most promising montage in this study. In the OFC-pre-SMA tDCS group, clinician-rated OC symptoms significantly decreased by a mean of −20.75% immediately after the 20th tDCS session, and the improvement remained 1 week and 1 month after tDCS. The results were similar for the self-rated OC symptoms as well. Moreover, our findings provide neurophysiological evidence that EEG beta-2 band functional connectivity within the frontal network (i.e., between the right and left superior/middle frontal gyrus) was significantly increased at the end of OFC-pre-SMA tDCS. Previous EEG research has indicated decreased EEG-lagged non-linear coherence at the beta-2 frequency within the frontal brain regions of OCD patients in comparison to healthy individuals [35]. Non-linear coherence represents a synaptic plasticity-associated basic process of neural communication, while non-linear coherence at the beta frequency is especially vital for the encoding and retrieval of memory as well as information maintenance [35]. A lack of synchronization of the oscillatory cycle of neuronal populations in the frontal network reflects altered intra- and inter-hemispheric neuronal communication within these areas during rest and may imply a patho-mechanism related to OCD. For other psychiatric disorders, such as bipolar disorder, depression, and schizophrenia, a novel ternary pattern-based automatic signal processing model is available for better classification performance [40]. Taken together, the findings reported herein are helpful for developing novel neurobiologically based intervention strategies for OCD. Future RCTs are needed to investigate whether OFC-pre-SMA tDCS improves OC symptoms through rectifying abnormal frontal network synchronization in patients with treatment-resistant OCD.

Our study shows that the Y-BOCS score decreased by a mean of −14.15% after the 20th session of cerebellum-OFC tDCS. Abnormalities in the cerebellum and OFC have been implicated in the pathophysiology of OCD. Neuroimaging studies in patients with OCD have reported hyperactive OFC, hypoactive cerebellum, and disconnectivity between the right and left cerebellar areas and between the OFC and the cerebellum [9]. tDCS targeting the right cerebellum and the left OFC has been reported as an efficient approach to alleviating symptoms in patients with OCD, possibly through its modulation of a large interconnected corticosubcortical network involved in the pathophysiology of OCD. By using head models for optimally targeting current delivery to structures of interest, earlier research on tDCS computational analysis revealed that tDCS using pre-SMA montage activated most of the areas related to OCD [41]. A recent study using electric field (EF) modeling analyses indicated that cerebellum-OFC tDCS montage could induce a stronger EF intensity in the right dorsolateral prefrontal cortex and bilateral SMA/pre-SMA than pre-SMA montage [9]. In a randomized sham-controlled double-blind study, patients with treatment-resistant OCD received 10 sessions of either active cerebellum-OFC tDCS (2 mA, 20 min, and two sessions per day) or sham stimulation [10]. The results indicated that cerebellum-OFC tDCS was not superior to sham stimulation in inducing a long-lasting reduction in symptoms (over a 12-week period) in patients with treatment-resistant OCD, although it induced a significant acute reduction in OCD symptoms immediately after the tDCS regimen compared to sham stimulation. Future studies are encouraged to investigate if a greater number of sessions (e.g., 30 sessions) with more extended periods of treatment (e.g., 6 weeks) can positively influence the efficacy of cerebellum-OFC tDCS in treating patients with treatment-resistant OCD.

The clinical benefit after cerebellum-OFC tDCS of the present study was also supported by the significant reduction in the EEG beta-1 band DMN connectivity between the region in the anterior cingulate gyrus and the right inferior parietal lobule. Aberrant DMN plays a crucial role in psychological processing related to pathophysiological mechanisms of OCD, and dysregulated DMN may contribute to aberrant self-referential processing and the preoccupation of self-oriented intrusive obsessive thoughts in OCD patients [42]. Neuroimaging studies have observed increased functional connectivity within the DMN in patients with OCD [43]. Similarly, increased DMN connectivity, which should be deactivated during cognitive task performance, was found when OCD patients received reward processing [44]. Regarding the inferior parietal lobule, another study showed OCD patients had higher DMN connectivity between the right inferior parietal lobule and the left ventral medial PFC. This connectivity, along with the anterior cingulate gyrus, is involved in emotional and reward processing and decision making [45,46]. Notably, both a thinned inferior parietal lobule and deficient neurite density in the right inferior parietal lobule among adult patients with OCD were reported [47,48]. It has been proposed that an inferior parietal lobule plays an important role in regulating internal thoughts and external information. A lack of cognitive flexibility related to dysfunction of the parietal cortex was found in patients with OCD. This leads to excessive focus on internally generated fears that are inconsistent with the evidence present in external cues [49]. In summary, the present study shows that cerebellum-OFC tDCS improved OCD symptoms after the end of stimulation. The results are supported by neurophysiological evidence of a reduction in the EEG beta-1 band DMN functional connectivity.

Notably, the Y-BOCS score only decreased by a mean of −7.4% after the 20th session of pre-SMA tDCS. Previous studies have reported that cathodal pre-SMA stimulation reduced OC symptoms measured by mean Y-BOCS severity scores more significantly than anodal stimulation, ranging from 20.1% (2 mA/20 min for 20 sessions) [50] to 26.4% (2 mA/30 min for 10 sessions) [51]. However, one double-blinded, randomized, sham-controlled study showed that excitatory anodal tDCS stimulation reduced OC severity by 22.06% [21]. The inconsistency among studies might be due to small sample sizes, lack of a sham group, the treatment duration, and variability in tDCS montage. For example, the study by Gowda et al., in which they used the anode over the left pre-SMA and the cathode over the right supra-orbital area, may have led to a different direction of the current flow, explaining the beneficial effect on reducing OC symptoms via excitatory anodal tDCS [21]. However, this does not explain the difference between our results and those from the study by D’Urso G et al. Both studies used the same montage, but a much lower reduction in OC symptoms occurred in the present study (7.4% vs. 20.1%). Another interesting finding of the present study is that the pre-SMA tDCS was the only montage to produce delayed tDCS effects, reducing the Y-BOCS scores by 14.4% and 22.8% in the one-week and one-month follow-up periods. It may take weeks to demonstrate efficacy using bilateral pre-SMA. The after-effects of tDCS have been linked to non-synaptic mechanisms involving neurogenesis [52]. It remains unclear whether the anode or cathode over the pre-SMA is the most suitable montage to treat OCD patients. Therefore, it is important to tailor the montage to each individual by applying EF modeling analysis, which accounts for interindividual anatomical variability. Additionally, a follow-up period is necessary to detect possible after-effects of tDCS, particularly for the pre-SMA region.

Our study has limitations. The first was the small sample size and the non-controlled, single-blind study design. The placebo response in the participants was not addressed because both the tDCS administrator and the patients were aware of the assigned treatment. The clinical response might have been inflated due to an expectancy bias. Nevertheless, a meta-analysis study with fifty-six trials demonstrated that OCD patients have a lower placebo response, with an average 10% improvement in their symptoms, compared to patients with other mental disorders (e.g., depression, generalized anxiety disorder), who experience a 25% or greater improvement while on placebo [39]. Second, the small sample size might have provided inadequate statistical power to detect significant differences in OCD symptom reduction among the three different intervention montages. However, the present study was hypothesis-driven to reveal that a novel dual-site cathodal tDCS treatment over the OFC and pre-SMA might be the most promising intervention for treatment-resistant OCD, accompanied by pathophysiological evidence from EEG. This study provides valuable research direction for patients with treatment-resistant OCD. Furthermore, we did not perform an ablation study to clarify the contributions of individual stimulation sites or their combined effect to validate the benefits of dual-site cathodal tDCS (OFC-pre-SMA) compared to individual stimulation sites. Third, this study did not enroll the target sample size. We enrolled 18 participants despite a targeted sample size of 36. We provide some reasons for the inadequate enrollment (please see Section 2.6. for the sample size calculation), but a potential type II error could not be totally excluded. The present findings may not be applied to the general population. Fourth, this study did not apply OCD symptom provocation before the tDCS sessions. The benefits of exposure therapies before repetitive TMS intervention have been revealed. Future studies are advised to investigate the benefits of OC symptom provocation in combination with tDCS. Furthermore, we did not examine the potential differential effects of tDCS on obsessions versus compulsions or other symptom dimensions. Fifth, EEG fails to detect sources of electrical activity in deep structures (e.g., the striatum, thalamus, and cerebellum) as well as the network connectivity in these areas. Furthermore, the EEG findings in the present study may not be conclusive due to the small sample size and lower statistical power to detect differences. Given the above-mentioned limitations, the present study provides implications for future research. A randomized three-arm, sham-controlled study with a larger sample size (e.g., at least 12 participants in each arm based on a previous sample calculation) is warranted to confirm our findings and examine the clinical efficacy of dual-site cathodal tDCS over the OFC and pre-SMA versus cathodal tDCS over the pre-SMA or the OFC (sham vs. dual-site of OFC and pre-SMA vs. OFC or pre-SMA) in patients with treatment-resistant OCD.

## 5. Conclusions

Due to the small sample size and lack of sham control, dual-site cathodal tDCS over the OFC and pre-SMA might be considered a potential montage to treat patients with treatment-resistant OCD. The present findings may not be generalized to other populations. We suggest that future studies examine the efficacy of this approach in larger samples of treatment-resistant OCD patients and explore the effects of concurrent dual-site cathodal tDCS over the OFC and pre-SMA (OFC-pre-SMA tDCS) in treating OC symptoms using randomized sham-controlled designs.

## Figures and Tables

**Table 1:** Timeline for treatment and assessments.

	Baseline	tDCS Intervention and Parameters *					End	1-Week Follow-Up	1-Month Follow-Up
**Outcomes**		D1	D2	D3	D4	D5	D12	D19	D40
		△ △	△ △	△ △	△ △	△ △			
		D8	D9	D10	D11	D12			
		△ △	△ △	△ △	△ △	△ △			
Y-BOCS	●						●	●	●
OCD-VAS	●						●	●	●
HAM-D	●						●	●	●
HAM-A	●						●	●	●
WHOQOL- BREF	●						●	●	●
CTT	●						●		
SCWT	●						●		
CPT-II	●						●		
TOL	●						●		
DSST	●						●		
HRV	●						●		
EEG	●						●		

* Twice-daily stimulation sessions were separated by at least 1 h and performed on 10 consecutive weekdays. Each session of tDCS consisted of a direct current of 2 mA delivered for 20 min. Abbreviations: D—day; tDCS—transcranial direct current stimulation; Y-BOCS—Yale–Brown Obsessive Compulsive Scale; OCD-VAS—Obsessive Compulsive Disorder Visual Analog Scale; HAM-D—Hamilton Depression Rating Scale; HAM-A—Hamilton Anxiety Rating Scale; WHOQOL-BREF—World Health Organization Quality of Life-BREF; CTT—Color Trails Test; SCWT—Stroop Color Word Test; CPT-II—Connors’ Continuous Performance Test, 2nd Edition; TOL—Tower of London–Drexel University Test, 2nd Edition (TOLDXtm); DSST—Digit Symbol Substitution Test; HRV—heart rate variability; EEG—electroencephalography; △ stimulation session; ● evaluation.

**Table 2:** MNI coordinates for the seeds from the default mode network (DMN) and frontal network (FN).

Network	Anatomic Structure	Side	XYZ (MNI)		
DMN	Anterior cingulate cortex (ACC)	Mid	0	53	0
	Posterior cingulate cortex (PCC)	Mid	0	−53	29
	Inferior parietal lobule (IPL)	R	60	−40	27
	Inferior parietal lobule (IPL)	L	−60	−40	27
FN	Anterior cingulate cortex (ACC)	Mid	0	41	0
	Superior/middle frontal gyrus (SFG)	R	31	36	38
	Superior/middle frontal gyrus (SFG)	L	−31	36	38
	Medial frontal gyrus (MFG)	R	14	48	−4
	Medial frontal gyrus (MFG)	L	−14	48	−4

Abbreviations: MNI, Montreal Neurological Institute; DMN, Default Mode Network; FN, Frontal Network. Notes: Coordinates are in MNI space. L = Left hemisphere seed; R = Right hemisphere seed; Mid = Midline seed. X = left (−) to right (+); Y = posterior (−) to anterior (+); Z = inferior (−) to superior (+).

**Table 3:** Sample characteristics at baseline.

Variables	Cerebellum-OFC tDCS	Pre-SMA tDCS	OFC-Pre-SMA tDCS	*p*-Value
Numbers	6	6	6	
Females (%)	2 (33.30%)	1 (16.70%)	2 (33.30%)	0.76
Age, years	37.67 ± 11.84	35.83 ± 13.32	30.67 ± 9.20	0.57
Education level, years	15.00 ± 1.10	14.83 ± 2.04	15.33 ± 3.72	0.94
Handedness (right/left)	0/6	2/4	0/6	0.11
Length of illness, years	18.67 ± 12.47	16.17 ± 11.20	13.83 ± 7.19	0.73
Comorbid medical conditions				
Type 2 diabetes	1 (16.70%)	1 (16.70%)	0 (0%)	0.57
Hypertension	1 (16.70%)	2 (33.30%)	2 (33.30%)	0.76
Dyslipidemia	1 (16.70%)	1 (16.70%)	0 (0%)	0.57
Current pharmacological treatment				0.55
Monotherapy with SSRIs or clomipramine, or combination of SSRIs and clomipramine	2 (33.30%)	2 (33.30%)	3 (50.0%)	
SSRIs or clomipramine				
augmented with an antipsychotic	2 (33.30%)	4 (66.70%)	3 (50.0%)	
Combination of SSRIs and SNRI	1 (16.70%)	0	0	
Combination of SSRIs and NDRI	1 (16.70%)	0	0	
Y-BOCS	31.67 ± 5.47	28.67 ± 6.74	26.00 ± 4.52	0.25
OCD-VAS	2.62 ± 2.06	3.67 ± 1.51	2.58 ± 1.02	0.43
HAM-D	10.67 ± 7.45	9.00 ± 5.10	7.00 ± 4.38	0.56
HAM-A	10.67 ± 6.41	8.00 ± 4.43	11.67 ± 8.57	0.63
WHOQOL-BREF				
Physical health	18.83 ± 2.64	18.17 ± 2.99	17.17 ± 3.31	0.63
Psychology	17.00 ± 2.19	14.83 ± 2.23	13.83 ± 2.48	0.08
Social relationships	10.67 ± 1.37	10.17 ± 1.33	9.67 ± 3.88	0.79
Environment	28.17 ± 2.04	30.00 ± 4.98	28.33 ± 6.80	0.79
TOL accuracy	4.50 ± 1.87	3.17 ± 2.64	2.83 ± 1.33	0.35
TOL time	233.17 ± 52.97	240.67 ± 77.59	233.67 ± 88.17	0.98
TOL score	1.00 ± 1.67	0.33 ± 0.52	0.50 ± 0.55	0.54
DSST	2.50 ± 2.17	3.17 ± 4.26	1.50 ± 2.74	0.51
CTT Color 1 time	45.64 ± 11.30	38.61 ± 14.50	40.41 ± 9.23	0.58
CTT Color 2 time	97.49 ± 40.00	91.88 ± 22.05	80.52 ± 15.14	0.57
CPT-II HRT	434.70 ± 56.06	401.84 ± 68.95	383.78 ± 78.38	0.45
CPT-II Var	11.56 ± 11.77	8.12 ± 6.37	6.82 ± 2.26	0.57
CPT-II d’	0.55 ± 0.25	0.49 ± 0.39	0.58 ± 0.52	0.92
SCWT Naming interference tendency	0.16 ± 0.15	0.44 ± 0.34	0.31 ± 0.35	0.28
SCWT Reading interference tendency	0.22 ± 0.15	0.44 ± 0.5	0.12 ± 0.05	0.21

tDCS—transcranial direct current stimulation; SSRIs—selective serotonin reuptake inhibitors; SNRI—serotonin and norepinephrine reuptake inhibitors; NDRI—norepinephrine and dopamine reuptake inhibitors; Y-BOCS—Yale–Brown Obsessive Compulsive Scale; OCD-VAS—Obsessive Compulsive Disorder Visual Analog Scale; HAM-D—Hamilton Depression Rating Scale; HAM-A—Hamilton Anxiety Rating Scale; WHOQOL-BREF—World Health Organization Quality of Life-BREF; TOL—The Tower of London–Drexel University Test, 2nd Edition (TOLDXtm); DSST—Digit Symbol Substitution Test; CTT—Color Trails Test; CPT-II—Connors’ Continuous Performance Test, 2nd Edition; d’—detection; HRT—hit reaction time; VAR—variability; SCWT—Stroop Color Word Test.

**Table 4:** Treatment-emergent adverse events among different tDCS groups.

Treatment-Emergent Adverse Events	Cerebellum-OFC tDCS	Pre-SMA tDCS	OFC-Pre-SMA tDCS	*p* Value
Daytime Sedation	3 (50.0%)	4 (66.7%)	5 (83.3%)	0.47
Numbness	2 (33.3%)	2 (33.3%)	2 (33.3%)	1.00
Inattention	2 (33.3%)	1 (16.7%)	0 (0.0%)	0.30
Burning sensation under the electrodes	1 (16.7%)	2 (33.3%)	0 (0.0%)	0.30
Pricking under the electrodes	0 (0.0%)	0 (0.0%)	2 (33.3%)	0.11
Dizziness	0 (0.0%)	0 (0.0%)	2 (33.3%)	0.11
Excitement	0 (0.0%)	0 (0.0%)	1 (16.7%)	0.35
Squeeze under the electrodes	1 (16.7%)	0 (0.0%)	0 (0.0%)	0.35
Itchiness under the electrodes	0 (0.0%)	1 (16.7%)	1 (16.7%)	0.57
Headache	1 (16.7%)	0 (0.0%)	0 (0.0%)	0.35
Acute mood changes	1 (16.7%)	0 (0.0%)	0 (0.0%)	0.35
Parageusia	1 (16.7%)	1 (16.7%)	0 (0.0%)	0.57
* Side effect score	0.40 ± 0.46	0.47 ± 0.64	0.33 ± 0.38	0.89

Notes: Each number represents the number (and percent) of participants who reported any given side effect at least once after the first session of stimulation. There were no significant differences between the occurrences of treatment-emergent adverse events in the three tDCS groups. * Side effect score was calculated using a score of 1 for each side effect reported by a participant in each session of tDCS. Data are presented as means ± standard deviations. The side effect scores were not significantly different between the three groups.

## Data Availability

The datasets generated during and/or analyzed during the current study are available from the corresponding author upon reasonable request.
